# Exogenous intoxication by non-prescribed use of vitamin D, a case report

**DOI:** 10.1186/s12877-020-01614-8

**Published:** 2020-06-24

**Authors:** Ana Laura Teodoro de Paula, Wemerson Philipe Ferreira Gonzaga, Lucas Martins Oliveira, Taciana Carla Maia Feibelmann, Juliana Markus

**Affiliations:** 1 Medical Doctorate Degree, Faculty of Medicine, Federal University of Uberlândia, Av Pará, 1720, Uberlândia, Minas Gerais 38405-320 Brazil; 2 Clinical Hospital of the Federal University of Uberlândia, Uberlândia, Minas Gerais Brazil; 3 Internal Medicine Department, Faculty of Medicine, Federal University of Uberlândia, Uberlândia, Minas Gerais Brazil

**Keywords:** Intoxication, Vitamin D, Hypercalcemia, Case report

## Abstract

**Background:**

This case report, unlike the current literature related to vitamin D intoxication, aims to highlight the risk of self-medication, and how publicity boosts the acquisition of vitamins for different purposes, increasing consumption with no professional indication or supervision. This practice can pose a serious health risk to the population.

**Case presentation:**

Our patient, a brazilian retired 64-year-old female, presented to the emergency service with post-prandial food vomiting of undigested content and stabbing abdominal pain with worsening during palpation. Concomitantly, onset of sporadic frontal headache, fatigue, hyporexia, weight loss of 18 kg in the same period, severe pruritus, musculoskeletal pain in the limbs and nocturia. The physical examination showed hypertension (160/80 mmHg) and itchy macules in the lower limb. Initially, the main diagnostic hypotheses were multiple myeloma, hyperparathyroidism and pancreatitis secondary to hypercalcemia, osteolytic neoplasms and other neoplasms that present with hypercalcemia. However, blood count, parathyroid hormone, chest X-ray, immunoglobulins, myelogram and bone marrow biopsy were not compatible with these diagnoses. Meanwhile, 25 OH vitamin D dosage and diluted vitamin D test confirmed the diagnosis of hypervitaminosis D. Hypercalcemic crisis was managed with vigorous hydration (50 ml/kg in 2 h), furosemide, bisphosphonates and blood pressure control with amlodipine and atenolol. Subsequently, the patient was discharged from the outpatient clinic with complete remission of symptoms, weight gain, serum calcium values of 10.76 mg/dL and ionizable calcium values of 6.52 mg/dL.

**Conclusion:**

Our report summarizes the possible consequences of using a vitamin compound without supervision of a competent professional, as these substances are mistakenly considered non-toxic. To add, little information is available about the supplements’ metabolism and their biological effects. Therefore, It is difficult to diagnose intoxication. This case report shows that even the self-administration of a product designed to bring health benefits can become a risky behavior. These vitamin and mineral supplements are supposed to bring patient empowerment and reduce government spending in health-care, but indeed represent a significant public health concern due to possible overdose and drug interactions.

## Background

This case report, unlike the current literature related to vitamin D intoxication, aims to highlight the risk of self-medication, and how publicity boosts the acquisition of vitamins for different purposes, increasing consumption with no professional indication or supervision. Therefore, this practice can pose a serious health risk to the population.

In the last years, speculations related to the possible extra-bone reactions of this hormone have been arising through controversial results of many different studies. These articles associate low levels of calcitriol with cancer, cardiovascular, metabolic, infectious and autoimmune diseases, beyond increased mortality. The proponents affirm that these associations establish a relation of cause and effect because there is biologic plausibility, since is well-known that the vitamin D receptor also regulates genes associated to immunity, cell differentiation and proliferation. The receptors can also be found in several tissues not involved with bone metabolism. However, none of the studies confirmed these presumptions [1].

In spite of non-conclusive evidences, population has been mistakenly informed that the use of supraphysiological doses of vitamin D can bring miraculous outcomes. Thereupon, the demand for its supplementation has increased either by prescriptions made by doctors or nutritionists, and also by frequent media orientations not based in scientific evidences [2].

Nevertheless, we must bear in mind that there are risk groups for hypovitaminosis D which require its supplementation. In these groups, dosing and supplementation is recommended to establish physiological body levels of cholecalciferol. The elderly, for example, are responsible for 43,4% of the outpatient cases and 71,2% of hospitalizations by lack of vitamin D, requiring supplements ingestion. Besides that, according to World Health Organization, newborn infants are at an elevated risk of vitamin D deficiency. Thus, vitamin D supplement to exclusive breastfed children is also highly recommended, aiming primary prevention of vitamin D deficiency and rickets [3–5].

However, the supplementation should be avoided in the general population by lack of conclusive evidence about the benefits of this practice. As exceptions, we should cite specific places lacking sunlight or with reduced exposure to sunlight due to staying inside or wearing clothes, what happens specially during harsh winters. And even for those groups for which supplementation is recommend, It should be made under supervision. Unfortunately, in Brazil, this recommendation hasn’t been followed, since a study conducted in 2016 showed a prevalence of 16,1% in self-medication, mainly composed by substances for which over-the-counter sales are allowed, the case of vitamin D [6].

According to international guidelines, the necessary daily dietary value for people up to 50 years old is 5mcg, regardless of sun exposure. For people aged 50 to 70, in turn, the value is 10 mcg and for people over 71 years old the value is 15 mcg [7]. The target serum level of vitamin D for the healthy population up to 60 years old is 20 ng/ml and for risk groups it is 30 ng/ml up to 60 ng/ml. In the risk groups, we highlight pregnant and lactating women, elderly, people with rickets/osteomalacia, osteoporosis, history of falls and fractures, hyperparathyroidism, inflammatory diseases, autoimmune diseases, chronic renal injury and malabsorption syndromes. Values above 100 ng/ml constitutes high risk of toxicity and hypercalcemia [4].

In addition, it’s generally unknown to the general population that this vitamin is stored in fat tissue for long periods. Hence, its indiscriminate use can disrupt calcium metabolism, creating an aggressive hypercalcemia, which causes lots of adverse effects in almost every body system. Hypervitaminosis D can lead to clinical signs such as persistent vomiting, weight loss, dehydration, psychiatric disorders and, at levels generally greater than 150 ng/ml, hypercalcemic crisis with difficult to control hypertension and acute kidney injury, setting a medical emergency that can lead to death [8].

We report a case of severe vitamin D poisoning through self-medication, pointing to the importance of raising this hypothesis as a differential diagnosis of hypercalcemia. In addition, we warn about the risk of using high doses, either by prescription or not, based on media apology for the need to maintain supraphysiological levels, leading to indiscriminate consumption by the population.

## Case presentation

A retired brazilian 64-year-old female started to present, 9 months ago, with postprandial vomiting of undigested content and stabbing abdominal pain. Those were constant symptoms that persisted for 12 months. Concomitantly, onset of lower limbs musculoskeletal pain, sporadic frontal headache, fatigue, hyporexia and weight loss of 18 kg in the same period. Few days after the onset of the abdominal and musculoskeletal pain, the patient reported nocturia, with 4 to 5 episodes each night with a foamy urine. The bowel habit alternated between constipated and normal, although there was no change in the appearance of the stool during the period of the disease.

The family history showed no significant informations beyond the low socioeconomic level. There were no significant past interventions related to the current symptoms.

**Fig. 1:**
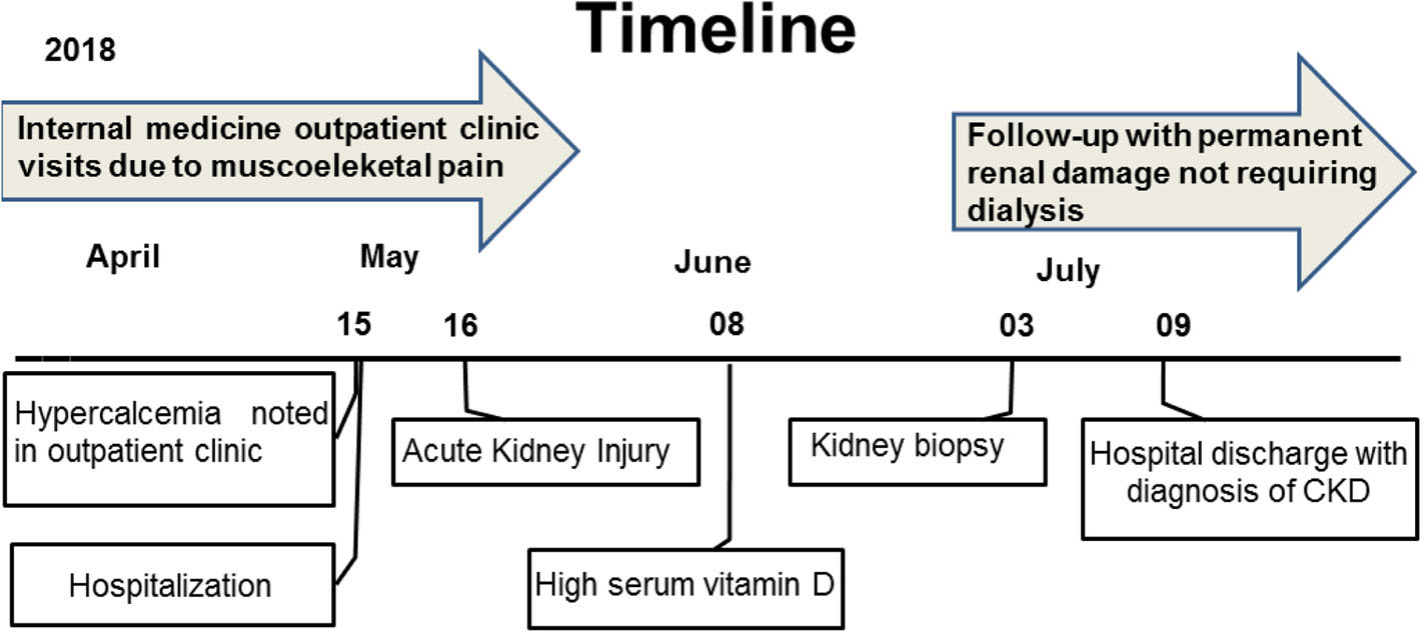
Timeline of the case

In the investigation of the condition, laboratory tests were requested (complete blood count, electrolytes, calcium, magnesium and pancreatic enzymes). The results showed a significant hypercalcemia and the patient was hospitalized, as we can see in the Fig. 1, which shows the disease timeline. At the time of admission, she was in good general condition, with diffuse cutaneous pallor, hypotrophic and eutonic muscles, discrete lower limb edema, capillary refill time of less than 3 s, anicteric, acyanotic and afebrile. She had a heart rate of 82 beats per minute, blood pressure of 160/80 mmHg and was eupneic. There were no abnormalities in the respiratory and cardiovascular examination. In the abdominal deep palpation, it was possible to notice mesogastric pain. It’s also important to take note of telangiectasias in the malleolar region of both limbs, and hypochromic and hyperchromic lower limb macules measuring up to 0.5 cm which, according to the patient, were local and severely itchy.

At the time, the main diagnostic hypotheses were multiple myeloma, hyperparathyroidism, pancreatitis secondary to hypercalcemia, osteolytic neoplasms and other cancers that present with hypercalcemia.

More laboratory tests were made, showing a normocytic and hypochromic anemia (Hb: 8.2 g/dL; Ht: 24.2%; MCV: 84.9 fL), leukocytes of 61,000/mm^3^ and platelets of 23,600/mm^3^. Also a serum calcium of 11.67 mg/dL and ionizable calcium of 6.51 mg/dL. Parathyroid hormone (PTH) was decreased (13.58 pg/mL) and there were high levels of urea (101.7 mg/dL) and creatinine (3.97 mg/dL), setting up a framework of acute kidney injury (Fig. 1) which lasted 2 months, not requiring renal replacement therapy.

Following the investigation of multiple myeloma and other pathologies, it was found an unchanged chest radiograph, normal values of immunoglobulins (IgA, IgG, IgM and total IgE), a normal myelogram and bone marrow biopsy showing only dysplastic abnormalities, not consistent with multiple myeloma.

Not confirming the previous diagnostic hypothesis, the team decided to perform the 25-hydroxy vitamin D dosage, which showed high serum values (100 ng/dL), as well as the diluted vitamin D test, which also presented high values (374 ng/dl), confirming the diagnosis of hypervitaminosis D.

Despite denying the use of non-prescribed drugs, when asked specifically about vitamins, the patient reported taking various vitamin complexes, including cholecalciferol once daily for about 6 months. She had access to several of them through samples provided by a close relative. Thus, exogenous vitamin D intoxication was diagnosed to the team’s surprise.

The team maintained hypercalcemic crisis management with pharmacological measures, in addition to prescribed paroxetine hydrochloride (20 mg once daily) to relieve the patient’s anxiety. The procedures performed were mainly vigorous hydration (50 ml/kg in 2 h), furosemide, bisphosphonates and blood pressure control with amlodipine and atenolol. Related to hypervitaminosis, behavior measures were conducted, with immediate cessation of supplementation use.

In July (Fig. 1), the patient was discharged from hospital in remission of musculoskeletal and gastrointestinal symptoms, weight gain, serum calcium values ​​of 10.76 mg/dL and ionizable calcium values of 6.52 mg/dL. According to serum calcium, we could access the adherence to behavioral measures, with complete cessation of vitamin D use. However, she was discharged with permanent renal damage. A serum creatinine of 1,77 mg/dl for a period of more than 3 months was noted on follow-up, closing a diagnosis of chronic kidney disease class KDIGO 4.

## Discussion and conclusions

Our report shows the case of a patient which suffered vitamin D toxicity by using a drug without a Dr. prescription and medical supervision. The investigation was prolonged by the variety of nonspecific symptoms presented by the condition and its rarity in relation to other more frequent pathologies such as neoplasms. These factor led to a delay in the beginning of adequate management. Another limitation of this case report consisted in the limited data contained in the medical records, which restricted our analysis. However, the case were strengthened by the social and political discussion about self-medication, its implications and causes.

Although a rare cause of hypercalcemia, vitamin D poisoning tends to become more prevalent as high doses of exogenous vitamin D are becoming available and increasingly prescribed by doctors. The wide variety of therapeutic uses to these products, allied to their profitable market, boost the publicity and, consequently, the consumption of vitamins for different purposes, such as anti-ageing, anti-stress, prevention of diseases and promotion of health. However, improper use, misrepresentation of the quantity of vitamins in packaging and lack of supervision pose a serious health risk to the population [9].

In the literature, there are many case reports of hypervitaminosis D due to several causes: manufacturing errors [2, 10, 11], overdosing by patients or prescribers [12], over-the-counter use [13–16] and many others, with possible combinations of the problems. What becomes clear is the lack of inspection related to labels and real dosages contained in the formulations, as well as misinformation and lack of information even by the doctors themselves. In the case reports above-mentioned, the presence of a dose many times higher than stated on the label was a frequent problem, that was discovered just with laboratory analyses made after the diagnostic hypothesis of intoxication.

Therefore, we strongly suggest that these medications should be tested prior to administration and the actual dosage should be assessed in comparison to the label information, since it is frequently unreliable. Such measures should be enforced through the law and strict inspection by regulatory agencies, since misinformation on the label can constitute fraud and a threat to public health. In addition, there is a clear need for educational campaigns aimed at doctors and the general public on the risks of vitamin D misuse, as well as the correct indications of its supplementation.

Even though there is no popular consensus on self-medication and consumption of these products, as they aren’t considered like drugs, the consequences can be equally harmful. The definition of self-medication encompasses, beyond the use of drugs without any professional guidance, the use of over-the-counter drugs and any industrialized mineral and vitamin supplements [17, 18].

In this context, our report summarizes the possible consequences of using a vitamin compound without supervision of a competent professional, as these substances are mistakenly considered non-toxic. But we must alert: in high doses and/or consumed for long periods, many vitamins can be even lethal [19]. To add, little information is available about the supplements’ metabolism and their biological effects, making it difficult to diagnose intoxication [9].

Furthermore, three recent studies demonstrated that self-medication is more common among women, probably because they are more likely than men to recognise and express symptoms [18, 20]. And this information is crucial for the promotion of awareness campaigns, especially, but not only, within this public.

Due to the lack of such policies, we then witnessed the effects of self-medication: chronically, hypercalcemia can cause several side effects, especially neurological, gastrointestinal, and renal ones. Hypercalcemia is deleterious to the function of excitable membranes, leading to musculoskeletal and smooth muscle fatigue. Effects on cardiac muscle include QT interval shortening and increased risk of cardiac arrest at very high calcium levels. Neurological sequelae include depression, irritability and, with sufficiently high levels, coma. Hypercalcemia rapidly exceeds the renal capacity for calcium resorption, and calcium leaks into the urine, causing nephrolithiasis. Beyond that, high levels of serum calcium can cause nephrocalcinosis and severely impair renal function, as it does to other soft tissues. Hypercalcemia also causes dehydration by inducing renal resistance to vasopressin, leading to nephrogenic diabetes insipidus. Dehydration, in turn, leads to a correspondingly greater increase in serum calcium concentration [2, 8, 21]. Concluding, we can see that our patient presented with most of these abnormalities.

Hence, we can highlight as most important take-away lesson from this case that the self-administration, without any medical guidance, even of a product designed to bring health benefits, can be framed as risky behavior and represents a significant public health concern due to overdose and drug interactions.

## Patient perspective

On a follow-up interview, the patient described the experience of the disease like a real challenge, due to the long-time hospitalization and the initial hypothesis of because of the hypercalcemia and renal symptons. She also referred the received treatment as good and said she was happy about the outcomes.

One aspect that calls our attention is that she hasn’t showed any concern about the permanent renal damage, which lead us to think that even after all that time of hospitalization and several invasive interventions, she hasn’t understood the seriousness of the intoxication.

## Data Availability

The datasets used and/or analysed during the current study are available from the corresponding author on reasonable request.

## Abbreviations

Hb: Hemoglobin
Ht: Hematocrit
MCV: Mean Corpuscular Volume
PTH: Parathyroid hormone
CKD: Chronic kidney disease
